# The impact of maternal diabetes on the future health and neurodevelopment of the offspring: a review of the evidence

**DOI:** 10.3389/fendo.2023.1125628

**Published:** 2023-07-03

**Authors:** Kalliopi Rodolaki, Vasilios Pergialiotis, Nikoleta Iakovidou, Theodora Boutsikou, Zoe Iliodromiti, Christina Kanaka-Gantenbein

**Affiliations:** ^1^ First Department of Pediatrics, “Aghia Sophia” Children’s Hospital, Medical School, National and Kapodistrian University of Athens, Athens, Greece; ^2^ First Department of Obstetrics and Gynecology, Alexandra Hospital, National and Kapodistrian University of Athens, Athens, Greece; ^3^ Neonatal Department, Aretaieio Hospital, School of Medicine, National and Kapodistrian University of Athens, Athens, Greece

**Keywords:** gestational diabetes mellitus, offspring neurodevelopmental impairment, neuroinflammation, fetal brain, pregnancy

## Abstract

Maternal health during gestational period is undoubtedly critical in shaping optimal fetal development and future health of the offspring. Gestational diabetes mellitus is a metabolic disorder occurring in pregnancy with an alarming increasing incidence worldwide during recent years. Over the years, there is a growing body of evidence that uncontrolled maternal hyperglycaemia during pregnancy can potentially have detrimental effect on the neurodevelopment of the offspring. Both human and animal data have linked maternal diabetes with motor and cognitive impairment, as well as autism spectrum disorders, attention deficit hyperactivity disorder, learning abilities and psychiatric disorders. This review presents the available data from current literature investigating the relationship between maternal diabetes and offspring neurodevelopmental impairment. Moreover, possible mechanisms accounting for the detrimental effects of maternal diabetes on fetal brain like fetal neuroinflammation, iron deficiency, epigenetic alterations, disordered lipid metabolism and structural brain abnormalities are also highlighted. On the basis of the evidence demonstrated in the literature, it is mandatory that hyperglycaemia during pregnancy will be optimally controlled and the impact of maternal diabetes on offspring neurodevelopment will be more thoroughly investigated.

## Graphical Abstract

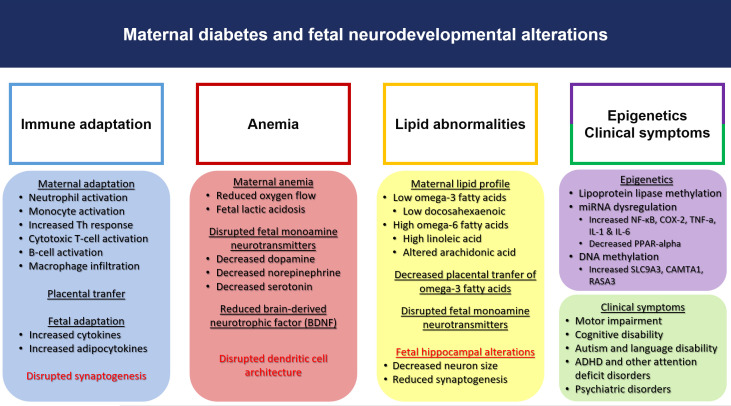

## 1 Introduction

Diabetes during pregnancy manifests as a disorder affecting the secretion and function of insulin, leading to hyperglycemia. This condition poses significant risks to the mother, the developing fetus, and the neonate, potentially resulting in severe morbidity. Diabetes can occur for the first time during pregnancy in a previously normoglycemic woman known as gestational diabetes mellitus (GDM). Alternatively, it may already exist as insulin-dependent (T1DM) or Type 2 diabetes mellitus (T2DM). It is estimated that approximately 14% of pregnancies worldwide are complicated by diabetes, making it one of the most significant challenges in obstetric care (1).

Diabetes during gestation is widely recognized as an established risk factor for multiple adverse outcomes in offspring from the neonatal period through adolescence and adulthood (2, 3). Maternal hyperglycemia during the perinatal period can result in vasculopathy, potentially leading to fetal hypoxia, fetal asphyxia, and even intrauterine death or spontaneous abortion (4). Fetal hypoxia may also stimulate increased erythropoietin production, resulting in polycythemia, hyperviscosity, an elevated risk of thrombosis, and hyperbilirubinemia. Additionally, maternal diabetes has been linked to the possibility of polyhydramnios, which can trigger placental abruption and premature delivery (5, 6).

Diabetic pregnancies have been associated with numerous neonatal complications. It has been observed that infants born to women with diabetes generally have lower Apgar scores and a higher incidence of neonatal hypoglycemia, particularly in cases of pre-existing diabetes (7, 8). In newborns, glucose serves as the primary energy source, especially for the central nervous system. Maintaining glucose levels within the normal range is crucial for proper brain function. Infants experiencing hypoglycemia may exhibit severe cerebral deficits, leading to future developmental delays and irreversible neurological damage, even in cases of mild hypoglycemia, especially within the first 6 h of life (9). Additionally, fetal hyperinsulinism can impede surfactant production, thereby delaying fetal lung maturation by interfering with the incorporation of choline into lecithin, which may contribute to the development of respiratory distress syndrome (10). Macrosomia (birth weight > 4 kg), resulting from fetal exposure to high insulin levels, the primary growth factor during intrauterine life, is observed in approximately 25% of diabetic pregnancies, leading to an increased risk of birth trauma (11).

Congenital defects are observed at a threefold higher rate in diabetic pregnancies, particularly when maternal diabetes is inadequately controlled (HbA1c > 7%) during the critical period of organogenesis in the first trimester (12). The risk of congenital defects is significantly elevated in pregnancies with pre-existing diabetes when glycemic control is inadequate (13). The precise pathophysiological mechanism linking maternal hyperglycemia to teratogenic effects is not fully understood. However, possible mediators include hyperketonemia, disrupted metabolism of arachidonic acid, myoinositol, prostaglandins, and hypoxia. Increased oxidative stress resulting from maternal hyperglycemia also plays a pivotal role in diabetic embryopathy, as it promotes cell apoptosis and modifies gene expression (6)

**Figure 1:**
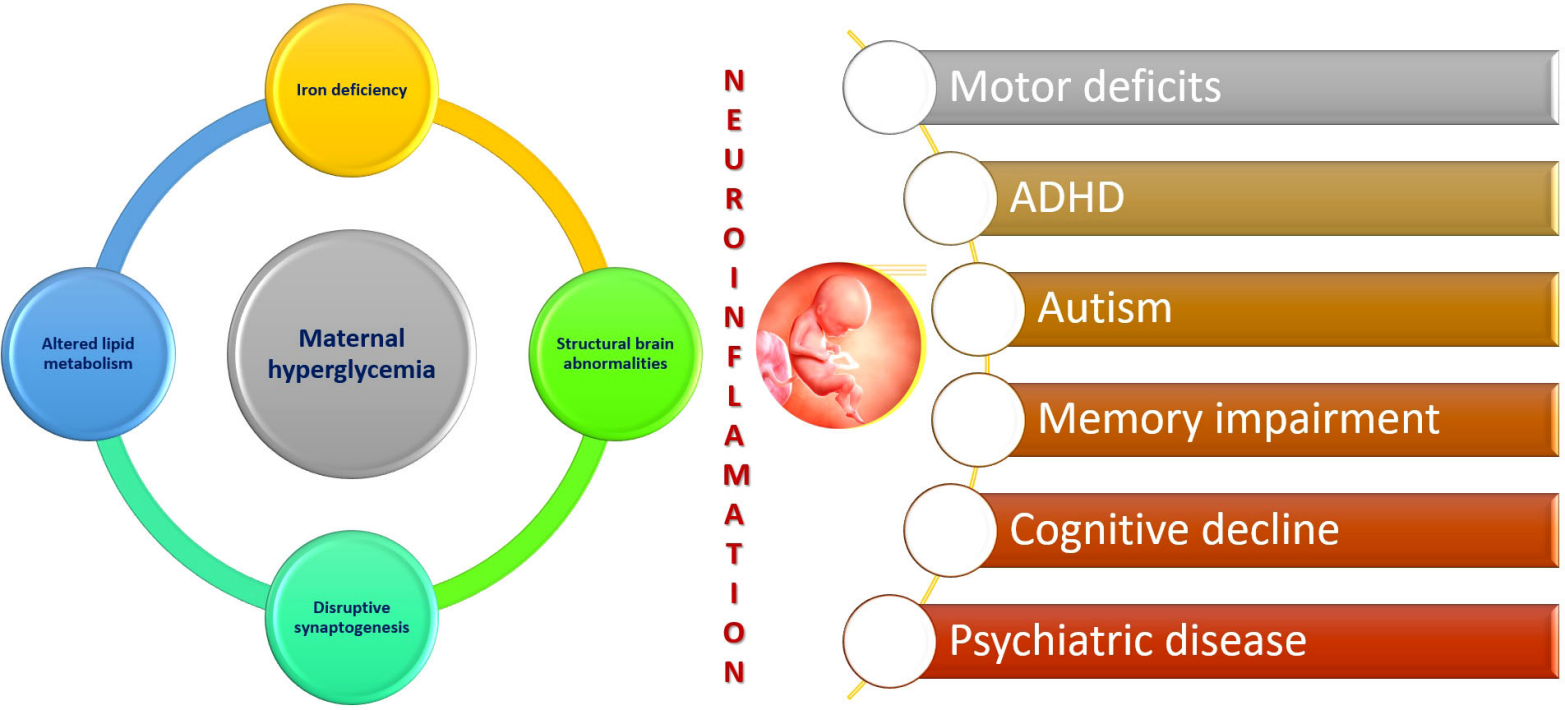
Maternal diabetes and neuroinflammation.

Subsequently, in later life, a growing body of evidence suggests that individuals born to diabetic mothers have an increased susceptibility to obesity, cardiovascular disease, metabolic syndrome, impaired glucose tolerance, T2DM, hypertension, and fatty liver disease (14). Furthermore, there is an increasing amount of data linking maternal diabetes to future neurocognitive and psychiatric disorders in offspring. The impact of exposure to a hyperglycemic intrauterine environment on neurodevelopmental outcomes in the fetus is still being investigated, with occasionally conflicting findings warranting further research in this field (15, 16). The intrauterine period is critical as fetal programming has been extensively demonstrated to affect brain development and function (17). Multiple mechanisms, including epigenetics, exaggerated inflammation and oxidative stress, and structural brain alterations, are gaining attention as potential factors underlying the influence of maternal diabetes on the developing brain (**Figure 1**).

Considering the above data, this review aims to underscore the maternal metabolic programming of neurodevelopmental impairments in offspring during hyperglycemic pregnancies and explore the potential underlying pathophysiological mechanisms through which maternal diabetes leads to adverse neurobehavioral outcomes. 

## 2 Materials and methods

For the present literature review, a comprehensive search was performed using the MedLINE, PubMed, EMBASE, and Scopus databases. Articles up to January 2023 were included, focusing on intrauterine exposure to diabetes and subsequent neurodevelopmental deficits. The following keywords were used alone or in combination: gestational diabetes, maternal diabetes, fetal brain, iron deficiency, epigenetics, neuroinflammation, neurodevelopment, motor impairment, cognitive impairment, attention-deficit hyperactivity disorder (ADHD), autism spectrum disorder (ASD), and psychiatric diseases. The bibliographic search for our narrative review was limited to publications written in English, and prospective, retrospective, case-control studies, reviews, and meta-analyses were included. Our review discusses and includes evidence from both human and animal subject.

## 3 Maternal immune activation and cytokine surge during hyperglycemia in pregnancy

Gestational diabetes constitutes a complex condition involving immune system dysfunction and a widespread inflammatory response, resulting in a surge of cytokines. Additionally, accumulating evidence suggests that neurodevelopmental disorders arise from an interplay between inflammation, immune dysregulation, and alterations in cytokines and pro-inflammatory molecules. Experimental studies indicate that diabetes during pregnancy induces a state of systemic inflammation, which exacerbates maternal immune activation. This immune activation is characterized by producing pro-inflammatory cytokines and chemokines, including various interleukins, tumor necrosis factor-alpha (TNF-alpha), RANTES factor, and eotaxin (49). Previous systematic reviews have shown that the activation of the maternal immune system during acute and chronic inflammatory conditions triggers the release of a significant amount of pro-inflammatory cytokines that can directly cross the placenta (50, 51). When these cytokines enter the fetal circulation, they can activate fetal immune cells, releasing molecules and cytokines like IL-6, which can cross the blood-brain barrier and promote fetal neuroinflammation. This pro-inflammatory state, characterized by elevated cytokine levels, synergizes with the toxic effect of hyperglycemia on the fetal brain, significantly disrupting central nervous system (CNS) maturation and resulting in severe neurodevelopmental consequences and psychiatric diseases (49). Moreover, maternal immune activation during pregnancy induces a significant imbalance between pro-inflammatory and anti-inflammatory cytokines in the fetal brain. Such alterations in cytokine expression within synapses may impede synaptic formation and function, ultimately affecting the stability of neuronal networks (50).

Obesity has previously been associated with a chronic inflammatory condition that significantly increases cytokine levels (52). Adipose tissue functions as a dynamic endocrine organ, releasing a substantial amount of adipokines, which possess both pro-inflammatory and anti-inflammatory properties. These bioactive molecules are synthesized and secreted by adipose tissue and, based on clinical study findings, exhibit various actions and effects, including notable changes in fetal physiology, such as alterations in fetal size, length, and adiposity (53). There is growing evidence regarding the role of adipocytokines in the pathogenesis of hyperglycemia-induced fetal consequences during pregnancy (54, 55). Clinical studies indicate chronic inflammatory diseases, including obesity and GDM, can disrupt the adipose-brain axis and contribute to cognitive impairment. It has been suggested that adipokines may regulate neuroplasticity and cellular metabolism in the brain, and their dysregulated expression has increasingly been linked to the pathophysiology of CNS dysfunction (56). Therefore, it is possible that adipocytokines, particularly pro-inflammatory ones like leptin, may mediate or worsen intrauterine neuroinflammation induced by impaired maternal metabolic conditions.

Leptin, a pro-inflammatory cytokine secreted by adipose tissue, has been extensively studied for its role in the CNS. In addition to its established role in metabolism and weight regulation, leptin has recently emerged as a potential mediator of neurodevelopmental disorders. Clinical studies have reported elevated leptin levels in infants of diabetic mothers and have correlated hyperleptinemia with the development of insulin resistance (57, 58). A study conducted using a large Boston Birth Cohort sample highlighted hyperleptinemia in children with ASD (59). More recently, Iwabuchi et al. analyzed data from 762 children participating in the Hamamatsu Birth Cohort for Mother and Child to explore the relationship between cord blood leptin levels, maternal metabolic conditions, and the future development of autistic behavior in offspring. Their findings indicated that although there was no apparent association between maternal metabolic disturbances and ASD development, there was a strong correlation between maternal diseases and cord serum leptin levels.

Furthermore, cord serum leptin levels were associated with specific subdomains of ASD, such as social interaction and repetitive behavior patterns in children assessed at 8–9 years of age. Thus, maternal diabetes may lead to impaired developmental outcomes through alterations in leptin signaling and other adipose tissue-derived cytokines (60). These effects are supported by previously published preclinical studies that have provided evidence of the neuroprotective function of leptin, involving the regulation of astrocytes (glial cells involved in homeostatic control and neuroprotection), oligodendrocytes (myelinating cells of the CNS), and microglia (CNS macrophage cells acting as the first line of active immune defense) (61).

## 4 Gestational diabetes: diagnosis, management and neurodevelopmental implications

The diagnosis of gestational diabetes is typically established based on the results of the Oral Glucose Tolerance Test(OGTT) which is routinely performed at 24–28 weeks of gestation. Maternal insulin resistance usually increases in the second trimester of pregnancy and cannot be identified before this time (62). While the OGTT test remains the gold standard for diagnosing gestational diabetes in most countries worldwide, there is an ongoing scientific debate regarding the optimal screening time and glucose thresholds (63). It remains uncertain whether an earlier diagnosis of gestational diabetes could potentially eliminate the potential consequences for both the mother and the developing fetus. Indeed, the results of several longitudinal studies investigating this matter have indicated that elevated glucose levels and resulting fetal hyperinsulinism have already developed long before a typical diagnosis of gestational diabetes is made, leading to fetal overgrowth and subsequent neonatal macrosomia (64, 65). Adverse perinatal outcomes, including accelerated fetal growth, appear to be significant in high-risk women (e.g., those with preconceptional obesity and a history of gestational diabetes). Thus, an earlier screening strategy should be strongly considered in these cases (66, 67).

Regarding the neurodevelopmental outcome of offspring of diabetic mothers (ODMs), evidence from previous studies has demonstrated that pre-GDM has the strongest effect on childhood neurodevelopmental impairment, reflecting the detrimental effects of exposure to a hyperglycemic environment on the developing brain from the early stages of pregnancy. Results from a recent birth cohort in Taiwan showed that all types of maternal diabetes, but especially T1DM, were associated with a higher risk of future development of neurobehavioral disorders (68). Therefore, it is reasonable to assume that although gestational diabetes only emerges during pregnancy, an earlier diagnosis of gestational diabetes could potentially benefit the developing brain by implementing an earlier strategy for maternal glycemic control. This way, the fetus would not be exposed to exaggerated glucose levels and resulting neuroinflammation during the critical early stages of brain development.

The first-line treatment for hyperglycemia during pregnancy is lifestyle adjustments involving physical activity and a customized diet plan. Physicians may need to implement pharmacotherapeutic interventions in refractory cases where maternal glucose levels are difficult to control. Several treatment strategies have been proposed, with insulin considered the gold standard for managing gestational diabetes (69). Among the different treatment options, insulin cannot cross the placenta, unlike oral antidiabetic drugs such as metformin or sulfonylurea glyburide, which may pass the placental barrier. However, evidence regarding these drugs’ direct effects on the developing fetus and long-term health outcomes remains limited (70). Moreover, metformin can cross the blood-brain barrier, raising questions about future neurodevelopmental outcomes after intrauterine exposure to the drug (71).

Evidence related to the extent of gestational diabetes control on the neurodevelopmental outcomes of offspring and differences between insulin and metformin treatment is derived from a limited number of human studies, and it appears that even in well-controlled cases, gestational diabetes results in minor neurological deficits. Most studies focusing on the future neurodevelopment of children exposed to metformin or insulin reveal that both regimens are equally effective, with no significant discrepancies in neurodevelopmental outcomes. The results of a follow-up of a randomized study comparing metformin and insulin in gestational diabetes revealed that 97 children at the age of 18 months had similar neurodevelopmental performances in both motor and language domains regardless of exposure to either insulin or metformin (72). Similarly, the study by Tertti K showed that the ODMs tended to poorer language performances, regardless of whether they received insulin or metformin treatment during pregnancy (73). These findings agree with the study by Wouldes et al., who observed no differences in neurodevelopmental outcomes between the two treatment approaches during gestation (74). Metformin seems to be a safe alternative to insulin, posing no significant neurodevelopmental risks. However, further research is warranted to focus on the differences between the various treatment modalities during pregnancy and their impact on the neurodevelopmental outcomes of the ODMs.

## 5 Maternal diabetes and the fetal brain

### 5.1 Iron deficiency

Accumulating evidence suggests that maternal diabetes affects the maturation of the developing CNS through various mechanisms. One of the most important factors influencing the developing fetal brain is considered to be diabetes-induced iron deficiency. Several clinical studies have highlighted that maternal diabetes leads to abnormal serum iron profiles in offspring, and this effect is highly correlated with maternal glucose levels (75–77). Gestational diabetes constitutes a state of fetal hypoxia with decreased oxygen supply, resulting in lactic acidosis, which, in turn, drives increased erythropoiesis and higher iron demands (78). Iron is well-known as a key transport mediator important for the proper oxygenation and function of all tissues in the human body, including the brain. The intrauterine period, especially the first trimester of gestation when organogenesis occurs, is a critical time window with high iron demands to adequately support the developing fetal brain, overall organogenesis, and the metabolically active placenta. Thus, iron deficiency can account for severe developmental deficits. Monoamine neurotransmitters like dopamine, norepinephrine, and serotonin, which are crucial for proper psychomotor function, require iron hydroxylases for synthesis. Alterations in the levels of these critical neuromodulators have been observed in states of iron deficiency, potentially leading to neurobehavioral consequences (79). Furthermore, experimental studies have shown that depleted fetal iron reserves may alter neuronal differentiation in the hippocampus, interfere with proper Brain-derived neurotrophic Factor (BDNF) function, and induce changes in dendritic cell architecture in animal brains. There also appears to be an association between iron deficiency and dysregulation in the expression of genes critical for brain function, neurobehavior, and synaptic plasticity (80, 81).

To date, there is limited clinical research investigating the extent of iron deficiency in diabetic pregnancies and its impact on future neurodevelopment. In the study by Sidappa et al., iron-deficient newborns of diabetic mothers exhibited weakened auditory recognition memory, which was already present at birth, as evidenced by the recording of Evoked-Related Potentials (ERPs). ERPs are brain responses to external sensory, motor, and cognitive stimuli that reflect coordinated neuronal activity. These same newborns were evaluated at 12 months of age using the Bayley Scales of Infant Development, which revealed defective motor performance (26). Similarly, in their study, Riggins et al. demonstrated that 3 to 4-year-old infants of diabetic mothers demonstrated specific deficits in recall memory that were proportional to the degree of prenatal iron deficiency (82). Therefore, the negative effects of depleted prenatal iron stores may persist beyond the neonatal period into childhood, potentially leading to significant developmental consequences.

### 5.2 Disordered lipid metabolism

All types of diabetes during pregnancy have been linked to disturbances in maternal lipid metabolism, consequently leading to compromised placental transfer of essential lipids in the developing fetus (83). Docosahexaenoic acid (DHA), an omega-3 fatty acid, is pivotal for the structure of neural tissue and retina, mitochondrial membranes, cerebral cortex, and the proper function of the brain. Animal studies have suggested that omega-3 fatty acid deficiency results in depleted DHA levels in the cerebral cortex, leading to learning disabilities (84). On the contrary, human studies have shown that DHA supplementation during pregnancy resulted in enhanced performance in problem-solving or sustained attention during infancy (85, 86).

As DHA cannot be synthesized by either the fetus or the placenta, the mother represents the primary supply of DHA to the fetus. In pregnancies complicated by diabetes, DHA transfer to the fetal circulation is impaired, leading to severe deficiencies that can, in turn, impair fetal neurocircuitry. Several mechanisms have been suggested to mediate the diminished placental transfer of DHA in pregnancies complicated by diabetes. Namely, peroxisome proliferator-activated receptor (PPAR)-alpha, a nuclear receptor responsible for genes related to fatty acid oxidation and transport, has been observed to be downregulated in the placenta of diabetic pregnancies compared to normal pregnancies (87).

Several studies have investigated the impact of dysregulated lipid metabolism in diabetic pregnancies and its effect on offspring neurodevelopment. The studies of Min et al. and Wijendran et al. demonstrated depleted levels of DHA in the cord blood of neonates of diabetic mothers compared to neonates of uncomplicated pregnancies (88, 89). A study by Zornoza-Moreno M et al. demonstrated that DHA levels were diminished in the cord blood of infants of diabetic mothers, and this reduction was further correlated with neurodevelopmental deficits at 6 months of age (90). Similarly, a recent prospective cohort study of 555 mother pairs provided evidence of impaired neurodevelopmental outcomes in infants of diabetic mothers during the first year of life, possibly mediated by alterations in lipid metabolism caused by maternal diabetes during gestation (91). The results of a recent meta-analysis of 24 observational studies confirmed that the cord blood of diabetic pregnancies has significantly lower levels of polyunsaturated fatty acids, potentially leading to severe neurocognitive sequelae for the exposed offspring (92).Taking these data into consideration, adequate supplementation of omega-3 fatty acids during pregnancy is mandatory, especially in cases of impaired lipid transfer to the fetus, such as maternal diabetes, in order to maintain sufficient distribution to both maternal and fetal circulation (93). Follow-up studies examining the long-term effects of omega-3 fatty acids on maternal and fetal metabolism will shed light on whether a higher intake of omega-3 fatty acids may benefit the future neurodevelopment of offspring in diabetic pregnancies.

### 5.3 Structural brain changes

#### 5.3.1 Impact of gestational diabetes on offspring’s brain structure and cognitive outcomes: insights from recent studies

Recent data from existing literature suggest that gestational diabetes promotes alterations in the structural morphology of the offspring’s brain. Van Dam et al. applied transcranial magnetic resonance studies to assess cortical excitability in 45 11–13-year-old children who were exposed *in utero* to GDM. These children showed depressed neuronal excitability and neuronal plasticity, suggesting that gestational diabetes may contribute to dysfunction of the CNS, which can be further correlated with impaired cognitive and motor outcomes (94).

In a recent study, researchers used diffusion tension imaging, a special MRI technique that estimates the organization of the brain’s white matter, and subsequently applied neurocognitive tests in infants of diabetic mothers and hyperglycemia-unexposed controls. According to their results, infants of mothers with GDM exhibited microstructural white matter abnormalities that were correlated with impaired neurocognitive performance (95).

In their study, Lynch et al. used magnetic resonance imaging to investigate the effects of gestational hyperglycemia on hippocampal architecture, as the hippocampus plays a crucial role in cognitive status and emotional regulation. It was observed that GDM induces a reduction in hippocampal size in specific sub-regions and causes sex-specific alterations in hippocampal morphology in middle childhood (96).

In another recent study, examiners investigated potential anatomical changes in 6-year-old children who had been exposed to gestational diabetes and obesity during pregnancy. The results indicated that children whose mothers developed gestational diabetes and had also gained excessive weight before pregnancy developed more profound structural brain abnormalities (97). Moreover, Ahmed et al. observed diminished cortical thickness in multiple brain structures and poorer overall cognitive performance in 9-year-old diabetes-exposed offspring (98).

#### 5.3.2 Effects of maternal diabetes on neurodevelopment in animal models: evidence from recent experimental studies

Several animal studies have revealed molecular and structural brain abnormalities in the ODMs (99). The most common way to create a diabetes model and investigate the effects of hyperglycemia during pregnancy in animal models is by using streptozotocin, a highly toxic agent for pancreatic beta cells, which leads to the destruction of insulin-producing cells and induces elevations in blood glucose levels (100).

Billy Vuong et al. induced GDM in rats by feeding them a diet rich in saturated fatty acids and sucrose and then assessed the cognitive status of their offspring. According to their findings, GDM-exposed offspring showed abnormal recognition memory and attention deficits, which were correlated with structural changes in the limbic system. Disordered development of hippocampal neurons, increased pro-inflammatory cytokine release in the brain, astrogliosis, and microglial morphological activation (suggested as a hallmark of CNS pathology and dysfunction) were all observed in the brains of GDM offspring (101).

Another study showed a remarkable reduction in hippocampal size and cellularity in the offspring of diabetic rats, which was not observed in control or insulin-treated rats. These morphological alterations indicate the susceptibility of neurons to increased serum glucose levels during neurodevelopment and may account for the learning and memory decline associated with maternal diabetes (102). Offspring of diabetic rats have also demonstrated increased apoptosis of hippocampal neurons, especially in sub-regions closely related to learning and memory processes (103). Several animal studies have specifically reported that elevated maternal glucose levels can lead to increased neuronal loss and interfere with active neurogenesis in the dentate gyrus and the CA1-3 pyramidal neurons of the hippocampus (104, 105).

Sousa et al. studied the offspring of pregnant rats with uncontrolled diabetes, who exhibited a remarkable delay in all fields of neurodevelopment due to their mothers’ uncontrolled diabetes. Interestingly, it was noted that maternal insulin therapy seemed to reverse the adverse effects of maternal diabetes, and male and female offspring reacted differently to the exposure to hyperglycemia, with male offspring demonstrating worse outcomes (106). On the contrary, a previous study had demonstrated that female offspring of diabetic rats exhibited increased susceptibility to hyperglycemic insults compared to male offspring, as indicated by suboptimal cognitive abilities (107). These results suggest that sex dimorphism exists, and offspring of different sexes should be separately evaluated and further studied concerning the effect of maternal diabetes on neurodevelopment.

In another rodent study by Chandna et al., the offspring of diabetic rats displayed behavioral abnormalities associated with increased excitability of hippocampal neurons. Moreover, their brains exhibited remarkable neuroinflammation, illustrated by enhanced receptor expression for advanced glycation end products (AGEs), commonly encountered in various CNS pathologies and psychiatric disorders (108). In a recent study, researchers evaluated the impact of diabetes on the brain by determining oxidative stress in specific brain locations, such as the cerebral cortex and the hippocampus. Elevated levels of reactive oxygen species, enhanced lipid peroxidation, abnormalities in glutathione metabolism, and a remarkable reduction in antioxidant brain levels were reported. These brain alterations have also been noted in patients with psychiatric and neurodegenerative diseases (109). Glutathione, known for its ability to reverse the damage created by reactive oxygen species (ROS), was found to be diminished in neonatal rats of diabetic mothers (110). Valle Bautista et al. observed defective neurogenesis and cell migration, impairments in dendritic branching, as well as alterations in the expression and location of several transcription factors vital for the optimal development of the CNS in the brains of rats born to mothers with diabetes (111). Finally, the results of a recent experimental study revealed depleted axonal neurogenesis in the fetal thalamus cortex and subsequent tactile sensory dysfunction in hyperglycemia-exposed rats (112).

### 5.4 Synaptogenesis

Several animal studies have suggested that maternal diabetes can impact the process of synaptogenesis, which is crucial for establishing brain connectivity and function. In rats, it has been observed that maternal diabetes induction leads to changes in the physiology of synaptophysin, an integral membrane glycoprotein found on presynaptic vesicles of neurons. Synaptophysin serves as a marker for synaptic density and proper synaptogenesis and is involved in learning and memory processes (113). In an animal model study, researchers discovered a significant reduction in synaptophysin expression in the cerebral cortex of neonatal rats born to diabetic mothers during the critical two-week period of synaptogenesis in the developing CNS (114). These findings align with another study conducted by Vafaei-Nezhad et al., which identified decreased synaptophysin expression in hippocampal neurons of newborn rats born to diabetic mothers after 1 and 2 weeks postnatally (115). Jing et al. also observed a decrease in synaptophysin levels in neonatal rats of hyperglycemic mothers, alongside a noticeable delay in fetal dendritic development in the brain. This delay may be attributed to the dysregulation of insulin and insulin-like growth factor I expression, both of which play significant roles in brain development, dendritic branching, and myelination (116). Additionally, Hami J et al. reported a decline in insulin-like growth factor 1 receptor levels in the cerebellum of rats born to diabetic mothers, supporting the notion that maternal diabetes has detrimental effects on the motor and cognitive development of offspring (117).

### 5.5 BDNF expression

Neurotrophins are a crucial family of peptides that play a significant role in developing the brain, peripheral, and central nervous systems. They are involved in neuronal growth, survival, and differentiation, possess antiapoptotic properties, and influence placental maturation. It has been proposed that neurotrophin levels may be downregulated in conditions characterized by increased oxidative stress. Cross-sectional studies have provided cumulative evidence showing that pregnancies complicated by diabetes exhibit higher levels of oxidative stress markers (such as 8-isoprostane and protein carbonyl) in both maternal and cord plasma. Consequently, an imbalance between oxidative stress and antioxidant defense mechanisms could potentially lead to altered neurotrophin levels in diabetic pregnancies. Omega-3 fatty acids are crucial in combating elevated ROS and maintaining normal neurotrophin levels. As mentioned earlier, impaired transfer of omega-3 fatty acids to the fetus has been observed in diabetic pregnancies, potentially resulting in deficiencies of these fatty acids and subsequent disturbances in neurotrophin regulation (118).

BDNF is a crucial neurotrophin that plays a role in neuronal differentiation, plasticity, and the establishment of synaptogenesis (119). Additionally, BDNF has been implicated as a mediator of glucose utilization and energy metabolism and has cytoprotective functions on pancreatic β cells. Decreased expression of BDNF has been observed in various neurodegenerative disorders characterized by neuronal loss, such as Parkinson’s and Alzheimer’s disease (120). Furthermore, low levels of BDNF have been reported in newborns who later developed ASD, suggesting its potential as a biomarker for neurodevelopmental disorders (121). In children with T1DM, particularly those with positive anti-GAD65 antibodies, lower serum BDNF levels and suboptimal neurocognitive performance have been observed (122). BDNF has also demonstrated neuroprotective effects against various adverse insults that threaten brain homeostasis, including cerebral ischemia, hypoglycemia, and GABAergic stimulations. Additionally, it enhances the action of neurotransmitters (123). Considering that gestational diabetes negatively impacts the neurobehavior of offspring and that adequate BDNF concentrations are crucial for optimal brain development, it is reasonable to assume that maternal diabetes may impair BDNF levels in offspring, leading to detrimental effects on their future neurocognition.

#### 5.5.1 Disruption of brain-derived neurotrophic factor (BDNF) expression in offspring due to maternal diabetes: implications for neurodevelopment

Normally, the hippocampus, which is crucial for proper memory processing, exhibits abundant expression of BDNF. However, a rodent study demonstrated that maternal diabetes leads to decreased BDNF expression, increased TNF-a concentrations, impaired cellular proliferation, and enhanced apoptosis in the offspring’s hippocampus, ultimately resulting in neurodevelopmental deficits (124). Consistent with other studies, a recent experimental investigation revealed significant dysregulation in the expression and density of BDNF in the hippocampus of offspring exposed to diabetes during critical periods of neurodevelopment (125). Additionally, reduced levels of hippocampal BDNF have been previously observed in mice born to obese mothers, and this reduction has been associated with cognitive impairment in the offspring (126). These findings underscore that metabolic disturbances during pregnancy can disrupt critical pathways of brain homeostasis.

#### 5.5.2 Exploring the association between BDNF levels and maternal diabetes in humans: recent observations and conflicting evidence

Limited human studies have been conducted on the association between BDNF and maternal diabetes. Briana et al. reported decreased BDNF levels in fetuses exposed to gestational diabetes, with BDNF concentrations being higher in girls than boys, potentially reflecting the incidence of neurodevelopmental disorders in male infants (127). In contrast, a recent prospective study investigating umbilical cord BDNF levels in pregnancies complicated by GDM found no differences between the GDM and control groups (128). Interestingly, reduced BDNF levels were observed in a small number of infants born to diabetic mothers at 12 months of age, and these lower levels were closely associated with impaired language development (129).

### 5.6 Epigenetic alterations

The current literature does not yet provide sufficient documentation on whether there is a connection between prenatal exposure to hyperglycemia and subsequent neurodevelopmental impairment through epigenetic modifications in gene expression. El Hajj et al. examined methylation levels of different genes in cord blood and placenta samples from the ODMs. They discovered significant alterations in genes previously associated with metabolism and obesity, implying that epigenetic changes might play a role in mediating the impact of maternal diabetes on the future development of metabolic disorders (130).

In neurodevelopment, only a limited number of experimental models have investigated the impact of maternal diabetes on gene dysregulation. In a recent study by Aviel Sheler et al., researchers observed behavioral abnormalities and impaired gene expression in the frontal cortex and striatum of male offspring from mothers with diabetes. The affected genes play a role in the proper formation of neuronal networks and, consequently, normal neurodevelopment (131). Another study identified the dysregulation of two genes encoding cytoplasmic proteins crucial for the process of apoptosis in the hippocampus of neonatal rats exposed to hyperglycemia. This group of rats also exhibited an increased expression of degenerating neurons. Dysregulation of programmed cell death has been proposed to be involved in various CNS pathologies, suggesting that the intrauterine hyperglycemic milieu may induce neuronal apoptosis dysregulation through epigenetic changes (132).

Similarly, Kandila et al. investigated the effects of hyperglycemia on DNA methylation in human neural progenitor cells and observed significant alterations in genes critical for neural tube development (133). Additionally, Zou et al. observed severe memory deficits in rats exposed to hyperglycemia and discovered epigenetic alterations in several genes associated with hippocampal synaptic plasticity (134). Finally, a novel study demonstrated the downregulation of genes expressed in the hippocampus of fetal mice exposed to GDM and alterations in hippocampal morphology. The differentially methylated genes are crucial in synaptogenesis, cognitive function, neuronal differentiation, neurotransmitter signaling, and dendritic development. Furthermore, significant changes in fetal brain metabolites associated with cognition and neurodevelopment, potentially attributed to DNA methylation changes, were noted (Graphical Abstract) (135).

## 6 Evidence linking maternal diabetes with impaired neurodevelopment of the offspring

Human epidemiological studies addressing the relationship between diabetes in pregnancy and neurodevelopmental deficits (**Table 1**).

**Table 1:** Human epidemiological studies addressing the impact of diabetes during pregnancy on offspring neurodevelopment.

SPECIFIC OUTCOME EXAMINED	STUDY	TYPE OF STUDY	SAMPLE	AGE GROUP	MAIN FINDINGS
Motor performance,	Churchill et al, 1969 (18)	Retrospective cohort	237 ODM, control group	8months old,12 months old	ODMs whose mother had ketonuria had impaired motor performance at 8 and 12 months and cognitive deficits at 4 years of age.
	Ornoy et al, 1999 (19)	Case-control	32 ODMs, 57 controls	5-12 years old	ODMs had Subtle Neuropsychomotor deficits especially in younger age and lower Verbal IQ than controls
	Ornoy et al, 2001 (6)	Case-control	57 from T1DM or T2DM, 38 from GDM,57 controls	5-12 years old	ODMs had more soft neurological signs which is associated with future inattention and poorer gross and fine motor performance, especially at 5-8 years and when the mother had higher HBA1ac or ketonuria.
	Saito Y et al, 2022 (20)	Prospective cohort	81,705 total, 2,162 ODMs.	6 months -4 years old	ODMs had significant developmental deficits especially in motor and problem solving domain
	Titmuss A et al, 2022 (21)	Prospective cohort	308 total -162 from women with GDM, 48 from women with T2DM	18–60 months.	ODMs displayed fine motor and problem solving deficits
	Torres-Espinola FJ et al, 2015 (22)	Case-control	313 children from mothers with obesity (64),GDM (79) overweight (56), controls (132)	8months old,12 months old	At 18 months of age,ODMs had impaired motor performance and deficits in expressive and composite language which was not observed at 6 months old.
	Ghassabian A et al., 2016 (23)	Prospective cohort	4909 total	4, 8, 12, 18, and 24 months of age.	ODMs had delayed gross motor milestone achievement compared to controls
Motor development	Lackovic M et al,2021 (24)	Prospective cohort	153 non-ODMSs, 50 ODMs	3 and 6 months	ODMs exhibited significant delays in motor development at 3 and 6 months of age
Memory function	Deregnier RA et al, 2000 (25)	Case-control	25 ODMs, 32 controls	Neonates	ODMs expressed slightly lower auditory memory performance than controls.
	Siddappa AM, et al, 2004 (26)	Prospective cohort	32 ODMs	12 months of age	ODMs had impaired recognition memory and developmental milestone deficits at 1 year of age
	Nelson CA et al, 2003 (136)	Case-control	11 ODMs, 16 control	8 and 12 months old	ODMs exhibited impaired recognition memory while their developmental milestones at 12 months were normal
Intellectual disability Academic performance	Leonard H et al, 2006 (27)	Prospective cohort	2686 ODMs	7 to 16 years old	ODMs had significantly greater rate of intellectual disability
	Dahlquist et al, 2007 (28)	Propsective cohort	6.397 ODMs 1.300.683 controls	Up to 16 years old	ODMs had a tendency of worst school performance than controls especially at the age of 16 years.
	Churchill et al, 1969 (18)	Retrospective cohort	237 ODM, control group	8months old,12 months old	ODMs whose mother had ketonuria had impaired motor performance at 8 and 12 months and cognitive deficits at 4 years of age.
	Ornoy et al, 1999 (19)	Case-control	32 ODMs, 57 controls	5-12 years old	ODMs had Subtle Neuropsychomotor deficits especially in younger age and lower Verbal IQ than controls
	Veena SR, et al, 2010 (29)	Prospective cohort	32 ODMs 483 control	9 years old	No association between lower cognitive abilities and GDM. On the contrary ODMs scored better than controls in certain tests
	Fraser A et al, 2012 (30)	Prospective cohort	8,515 total 26 with pre-existing diabetes, 33 GDM and 264 with glycosuria	4 years, 8 years and 16 years old	Diabetes during pregnancy was associated with worst school performance and lower offspring IQ
	Mann JR et al, 2013 (31)	Retrospective cohort	165.311 total, 17.988 ODMs	Birth-3 years old	ODMs had increased risk of developing intellectual disability, especially in pre-pregnancy diabetes cases although the association was not statistically significant
	Heldarskard GF et al, 2021 (137)	Prospective cohort	4286 ODMs,501.045 controls	15 years old	ODMs had worst academic course than children not unexposed to diabetes
	Soepnel LM, et al, 2022 (32)	Prospective cohort	95 offsprings of hyperglycaemic mothers, 99 unexposed offspring	3-6 years old	Hyperglycemia during pregnancy was associated with cognitive impairment in the offspring
	Cai S et al, 2016 (33)	Retrospective cohort	74 ODMs, 399 controls	6, 18 and 24 month	ODMs presented with electrophysiological brain changes at 8 and 18 months of age which have been associated with future inattentive behaviors.
	Krakowiak P, et al, 2012 (34)	Prospective cohort	1004 total	2-5 years old	ODMs with autism had a higher rate of cognitive and language deficits
Language ability	Torres-Espinola FJ et al, 2015 (22)	Case-control	313 children from mothers with obesity(64),GDM (79) overweight (56), controls (132)	8months old,12 months old	At 18 months of age,ODMs had impaired motor performance and deficits in expressive and composite language which was not observed at 6 months old.
	Dionne et al, 2008 (35)	Case-control	221 ODMs, 2612 controls	18 months old- 7 years old	ODMs had impaired expressive language scores at 18, 30, and 72/84 months old.Genetic factors and better maternal education seem also to affect the lesser the effects of maternal diabetes
	Sells C.J et al, 1994 (36)	Case-control	109 ODMs,90 controls	6,12, 24 and 36 months	ODMs had worst performance in language development than controls
Attention deficits ADHD	Bytoft B et al., 2016 (37)	Prospective cohort	269 ODMs with T1DM, 293controls	16-19 years old	No significant association between maternal Type 1 diabetes and attention deficits,higher rate of ADHD medication in ODMs
	Nomura Y et al, 2012 (38)	Prospective cohort	21 ODMs, 191 controls	4 and 6 years old	ODMs had a high risk of developing ADHD at 6 years of age especially when combined with low socioeconomic status
	Perea V et al, 2022 (39)	Prospective cohort	1036 ODMs	17 years old	ODMs had increased risk od developing ADHD especially if their mother had gained excessive weight during pregnancy
	Xiang AH et al, 2018 (40)	Retrospective cohort	37,878ODMs(522 exposed to T1D, 7,822 T2D, and 29,534 GDM)	Mean age 4.9 years old	Maternal diabetes wa associated with increased risk of ADHD
	Lin CH et al, 2019 (41)	Retrospective cohort	14 ODMs	6-10 years old	Higher rate od ADHD in ODMs especially male and those born full term.
	Perea V et al, 2022 (39)	Prospective cohort	1036 ODMs	17 years old	ODMs had increased risk od developing ADHD especially if their mother had gained excessive weight during pregnancy
Behavioral problems And Psychiatric disorders	Krzeczkowski JE. et al, 2019 (138)	Prospective cohort	586 total	2 years old	GDM and elevated pregnancy BMI was associated with externalising and internalising symptoms in the offspring although the association was not statistically significant
	Nahum Sacks M et al, 2016 (15)	Prospective cohort	231,271 total, 12642 GDM	N/A	ODMs had significantly higher rates of ASD, eating disorders, and obstructive sleep apneas
	Yamasaki S, et al, 2019 (42)	Propsective cohort	4478 Total	10 years old	ODMs had increased risk of having hallucinations
	Kong L,et al, 2020 (43)	Prospective cohort	647. 099 total, 4000 offsprings from insulin-treated diabetes, 3724 offsprings from T2DM, 98.242 offsprings from GDM	All ages until 19 years old	Maternal diabetes (especially type 2) together with maternal obesity was associated with increased risk for multiple psychiatric disorders especially mood disorders, ASD, and ADHD or conduct disorders.
	Noqueira Avelar E Silva, et al., 2021 (139)	Prospective cohort	56.206 ODMs (22614 from T1DM, 6.713 from T2DM, 26.879 from GDM	Until 40 years old	ODMs had increased risk of developing psychiatric diseases
	Sun G et al, 2022 (44)	Case-control	23 ODMs, 20 controls	Neonates	ODMs had decreased ability of emotional processing compared with controls
	Wang P et al, 2021 (45)	Propsective cohort	1036 total, 228 ODMs	12 months	ODMs had worst communication skills than non-ODMs
AUTISM	Chen S et al, 2021 (46)	Prospective cohort	2.369.680 offsprings	Up to 29 years old	Maternal diabetes was associated with increased risk for ASD,ADHD and intellectual deficits,especially Type 2 Diabetes mellitus and when the onset of GDM was at 27-30 weeks of gestation
	Persson M et al, 2022 (47)	Prospective cohort	1.406.650 TOTAL, 8003 from T1DM	4 years old	ODMs has increased risk of developing autism especially if born pre term
	Xiang AH et al, 2018 (48)	Retrospective cohort	419 425 total,621 exposed to T1DM, 9453 to T2DM, 11 922 to GDM diagnosed by 26 weeks’ gestation, 24 505 to GDM diagnosed after26 weeks’ gestation.	median follow-up of 6.9 years after birth	Maternal T1DM followed by T2DM and GDM to a lesser extent was associated with increased risk of autism in the offspring.
	Zhu B et al, 2021 (140)	Prospective cohort	3260 total, 419 ODMs	18 and 36 months of age	ODMs had higher odds of developing autism but not ADHD

### 6.1 Motor impairment

Several studies have examined the impact of maternal diabetes on the attainment of infant motor milestones. Most studies support an association between diabetes during pregnancy and poorer motor performance in offspring. As early as 1969, Churchill et al. reported that children born to diabetic mothers with ketonuria during pregnancy showed impaired motor scores at 8 months and postural control at 12 months. The study findings were unaffected by maternal insulin treatment during pregnancy, while children born to mothers without ketonuria showed no difference in neurodevelopment compared to controls (18). Later, Ornoy et al. utilized the Bruininks-Oseretsky Motor Development Test, which assesses fine and gross motor development in children aged 4.5 to 14.5 years. Their results suggested that the ODMs, both with pre-gestational and gestational diabetes, exhibited lower motor scores than the control group, particularly when their mothers had higher glycosylated hemoglobin levels or severe acetonuria (141). In the Upstate Kids Study, a population-based birth cohort study, mothers with various underlying medical conditions recorded their children’s milestone achievements at ages up to 24 months. The results indicated that infants of diabetic mothers experienced a slight delay in sitting, walking alone, standing, and walking with assistance (23). In 2015, Torres-Espignola et al. conducted a prospective study involving 331 mothers, and their children were assessed using the Bayley III Scales of Infant and Toddler Development at 6 and 18 months of age. At 18 months, children of obese mothers and mothers with gestational diabetes exhibited lower scores in gross motor development (22). Consistent with other researchers, Lackovic et al. also confirmed impaired motor performance at 3 and 6 months of age in infants exposed to GDM (24). Furthermore, a recent longitudinal birth cohort study by Titmuss et al. investigated the effects of T2DM and GDM on offspring neurodevelopment at 16–60 months of age and reported defects in fine motor performance among offspring exposed to hyperglycemia (21). In a systematic review and meta-analysis conducted by Diana Arabiat et al., including 13 studies, maternal diabetes, especially when present before pregnancy or coexisting with other comorbidities such as hypertension and obesity, was found to harm the motor development of offspring (142).

### 6.2 Attention-deficit hyperactivity disorder

Maternal diabetes is increasingly recognized as a potential risk factor for developing common neurodevelopmental disorders, including ADHD, in offspring. ADHD is one of the most prevalent neurodevelopmental disorders characterized by a persistent pattern of inattention, hyperactivity, and/or impulsivity, which disrupts a child’s normal development in various domains. Children diagnosed with ADHD often exhibit poorer school performance, difficulties in socializing, and an increased risk for substance abuse and other mental disorders (143). Multiple studies have explored the association between ADHD and maternal diabetes during pregnancy, yielding conflicting results. Ornoy et al. suggested in their study that school-age children exposed to maternal pre-gestational diabetes exhibited increased soft neurological signs during neurological assessments, indicating a higher likelihood of attention-deficit and/or hyperactivity (141). A recent population cohort study demonstrated that the diagnosis of GDM between 27 and 30 weeks of gestation is associated with a higher risk of developing ADHD.

Furthermore, the study suggested that the odds of ADHD and other neurodevelopmental disorders are greater when coexisting intellectual disability is present (46). A retrospective birth cohort study by Xiang et al. in 2018 reported that the offspring of mothers with GDM who required antidiabetic medication to lower their glucose levels during pregnancy had a higher likelihood of developing ADHD than children not exposed to hyperglycemia. Conversely, it was noted that GDM that did not require antidiabetic therapy did not pose an increased risk for ADHD. Additionally, the study indicated that, compared to normal pregnancies, the offspring of women with T1DM had the greatest risk for ADHD, followed by those with T2DM and, finally, those with GDM. Thus, the severity of hyperglycemia during pregnancy constitutes an independent risk factor for the development of ADHD in the next generation. However, the study found no association between the timing of diabetes diagnosis during gestation and ADHD (40).

In a prospective study conducted in Singapore, researchers reported electrophysiological alterations in 6- and 18-month-old children exposed to diabetes during pregnancy, particularly in the left hemisphere responsible for attention processing. These findings further support the idea that maternal diabetes may strongly impact the development of ADHD. Additionally, these alterations in neuronal activity were found to be associated with maternal blood glucose levels (33). Nomura et al. observed that when GDM is combined with low family socioeconomic status, there is approximately a 14-fold increased risk of developing impaired social functioning and ADHD symptomatology (38). Consistent with other researchers, a retrospective study from China indicated an increased incidence of ADHD in children of diabetic mothers, especially in males and full-term infants. Notably, gestational and pre-gestational diabetes posed a 2.6-fold increased risk for future ADHD development compared to the control group (41). Recently, Perea et al. conducted a prospective study to assess the association between maternal weight gain in pregnancies complicated by GDM and the future development of ADHD in offspring. Interestingly, the results indicated a higher rate of ADHD in the ODMs whose mothers had a combination of pre-gestational obesity and extreme weight gain during pregnancy (39).

However, a study by Bytoft et al. examined the effects of maternal T1DM on adolescent offspring, and the results did not reveal a higher rate of attention problems in the adolescent population. Nevertheless, there was a tendency towards increased use of ADHD medication among adolescents exposed to diabetes (37). Furthermore, a recent systematic review and meta-analysis investigating the impact of maternal diabetes on the most prevalent neurodevelopmental disorders found no significant association between maternal diabetes and ADHD, while a strong association was observed with ASD (144).

### 6.3 *Autism* spectrum disorders

To date, there has been a strong association between metabolic disturbances during the perinatal period and the development of ASD. ASD, also known as pervasive developmental disorders, is a group of disorders characterized by early-onset deficits in communication and social interaction and repetitive behavior patterns. ASD is considered a multifactorial disorder with genetic and environmental factors playing a role (145).

#### 6.3.1 Maternal diabetes and its implication in autism spectrum disorder development: evidence from recent epidemiological and prospective studies

A meta-analysis of case-control studies conducted in 2018 by Hongquan Wan et al. suggested a correlation between diabetes in pregnancy, particularly gestational diabetes, and an increased incidence of ASD in offspring (146). In the same year, Xiang et al. conducted a retrospective cohort study involving a substantial number of participants, which indicated that both T2DM and GDM diagnosed before 26 weeks of gestation, as well as T1DM, pose a significant risk for the future development of autism (48). Shuyun et al., also part of the aforementioned retrospective study, found that the risk of ASD in offspring was higher in the context of maternal T2DM (46).

In a recent prospective birth cohort study involving 1036 mother-child pairs, the relationship between GDM and neurodevelopment in offspring was assessed at 12 months of age using the Ages and Stages Questionnaire, a screening test for developmental delay in infants. The study revealed that GDM was associated with notably poorer scores in the communication domain, and this association may be mediated by elevated cord blood C-peptide levels. Furthermore, every 1 SD alteration in maternal glucose levels during gestation was associated with a higher risk of deficits in communication and personal social domains, which are closely associated with ASD in terms of neurodevelopment (45). Kong et al. reported a six-fold increase in the odds of ASD and ADHD development in children up to 11 years old whose mothers had pre-gestational diabetes and a higher body mass index (BMI) during pregnancy (147).

Another prospective study conducted in China demonstrated that gestational diabetes increased the development of autistic traits in toddlers when examined at 18 months of age and induced alterations in the expression of placental inflammatory markers such as TNF-a and IL-6. However, the disordered expression of these cytokines did not seem to mediate the effects of intrauterine hyperglycemia on autism development (140). Furthermore, the results of a novel study by Persson M et al. revealed that T1DM poses a significant risk factor for developing autism later in life, especially in children born prematurely (47). Additionally, according to another recent study, the ODMs displayed significantly poorer ability to discriminate emotional prosodies during the neonatal period compared with controls. Considering that children with autism often have difficulty expressing their emotions properly, these findings may indicate a greater risk of developing ASD in the ODMs (44).

#### 6.3.2 Animal studies elucidating the link between maternal diabetes and autism-like behaviors: mechanisms and potential interventions

The relationship between autism and maternal diabetes has also been explored in various animal studies. In a previous study by Wang et al., it was reported that increased oxidative stress induced by maternal hyperglycemia promoted autistic characteristics in rat offspring by inhibiting superoxide dismutase 2 (SOD2), an enzyme known for its antioxidant and protective effects against ROS. The repression of SOD2 was also associated with dysregulation of mitochondrial and fatty acid metabolism, which have previously been linked to autism development (148).

Another rodent study indicated that the offspring of hyperglycemic rats developed social deficits similar to those seen in ASD through epigenetic alterations in oxytocin receptors and increased oxidative stress (149). Similarly, an experimental study using female Wistar rats reported that neonatal rats born to mothers with diabetes exhibited autism-like behavior patterns. The offspring of diabetic rats showed disrupted gut microbiota, elevated levels of ROS and IL-6, and significantly decreased GABA levels, which are critical for emotional perception. Interestingly, this study found that insulin therapy given to diabetic rats appeared to counteract these pathophysiological mechanisms induced in the offspring, suggesting that insulin therapy during pregnancy may prevent autism-like behavior (150).

In a recent experimental study investigating autism development in the context of maternal diabetes, autistic behavior was also observed in mice born to mothers with diabetes. The negative effect of maternal diabetes seemed to be mediated by suppressing retinoic acid-related orphan receptor alpha (RORA) and further suppressing aromatase and SOD2. RORA is a nuclear receptor involved in multiple processes, including immunity and metabolism, and is reduced in ASD patients (151).

### 6.4 Memory function

Only a limited number of clinical studies have investigated the potential impact of maternal diabetes on the memory function of offspring. In the early 2000s, Deregnier RA et al. assessed the recognition memory of infants born to mothers with both gestational and pre-gestational diabetes using ERPs and identified slight deficits in memory recognition (25). In 2003, Nelson et al. evaluated cross-modal (tactile to vision) memory in infants of diabetic mothers compared to control children. The children exposed to diabetes showed impaired recognition of palpated objects, despite having normal Bayley scores (136). A longitudinal study conducted by Adeline Jabeen et al. in 2015 examined the effect of maternal diabetes on 19 children assessed at the age of 10 years. The study did not find any memory impairment at this age; however, when assessing hippocampal volume in these children – a brain region essential for memory development – they observed that higher hippocampal volume was associated with slower performance on certain tasks, which was an intriguing finding not observed in control children (152). In summary, the current literature regarding the effect of maternal diabetes on offspring memory function is limited, and further research is needed to address this knowledge gap.

### 6.5 Language development

Speech and language development are crucial in a child’s acquisition of normal milestones. These skills are primarily developed during the early years of life when the infant’s brain is highly responsive to external environmental stimuli. Developmental pediatricians commonly use multiple screening tests to evaluate both receptive and expressive language abilities.

In a study by Sells et al. (36), it was reported that at the age of 3, infants of mothers with poor diabetic control showed a reduction in verbal performance. However, there were no differences in verbal performance between controls and infants of mothers with optimal glycemic control. Another study conducted in Quebec in 2008 compared language development between infants of diabetic mothers (IDMs) and controls. The findings indicated that gestational diabetes hindered normal language evolution, as IDMs had lower vocabulary and grammar acquisition scores at 18 and 30 months. This study also revealed that infants of mothers with higher education were less affected in their language development, suggesting that genetic predisposition may also play a role (35).

Krakowiak et al. (34) assessed children of mothers with metabolic conditions, including diabetes, using the Mullen Scales of Early Development. They reported that these children performed worse than other children in the domain of receptive language ability. A meta-analysis was conducted to investigate the association between diabetes and language impairment, although only a limited number of studies on this subject were identified. The results of the included studies showed high heterogeneity, but it was suggested that diabetes might diminish language performance in children (153).

### 6.6 Intelligence quotient, learning*, and* academic performance

Intellectual development encompasses the dynamic process through which a child develops their ability to think, acquire knowledge, and organize it effectively for appropriate interaction with the social environment. However, clinical studies investigating the relationship between diabetes during pregnancy and intellectual disability in offspring are limited in the literature.

Churchill et al. (18) were among the first researchers to explore the impact of maternal diabetes on the future development of the child. They reported that children born to diabetic mothers with ketonuria during pregnancy exhibited cognitive deficits at 4 years of age. Ornoy et al. later reported poorer verbal IQ performance in young school-age children of diabetic mothers than in age-matched controls (19). A population-based study conducted by Helen Leonard et al. noted that maternal diabetes and other maternal medical conditions negatively affected the intellectual development of offspring (27). In 2013, Mann et al. found that pre-existing diabetes before pregnancy had a more significant negative impact on intellectual development than diabetes that developed during gestation (31).

Fraser et al. conducted a study using data from a prospective pregnancy cohort, assessing children born to diabetic mothers in terms of their IQ and academic performance at different ages. The results showed lower verbal IQ scores in children born to mothers with diabetes and glucosuria when assessed at 8 years old using the Wechsler Intelligence Scale for Children. The strongest association was observed with diabetes that developed during pregnancy. At 16 years of age, children born to women with pre-existing diabetes, and to a lesser extent, GDM, achieved poorer grades in their educational assessments (30).

In a recent study, researchers examined the cognitive performance of 3 to 6-year-old children of women with gestational hyperglycemia in an urban African population. They found significantly lower cognitive scores in children of hyperglycemic mothers than in children of euglycemic women. However, no notable association was observed between gestational hyperglycemia and the motor performance of exposed children (32). Additionally, a recent birth cohort study, which included 2,162 pregnancies complicated by GDM, assessed offspring neurodevelopment up to 4 years of age. The study found that diabetes-exposed offspring, especially males, displayed notable defects in problem-solving skills (20).

In contrast, a study from India revealed that a relatively small number of ODMs exhibited better learning, long-term storage/retrieval, and verbal ability at ages 9-10 when assessed using Kaufman’s Assessment Battery for Children and other tests. These discrepancies in findings may be attributed to variations in the tests and methods used to evaluate the cognitive abilities of the participants (29).

A recent study by Tingting Xu et al. reported that children of mothers with abnormal glucose tolerance at 26-28 weeks of gestation did not show clinically significant differences in cognitive and behavioral outcomes compared to control children during mid-childhood. This suggests that the impact of maternal diabetes on child cognition diminishes over time. However, the study also found a slightly reduced total score in the Wide Range Assessment of Visual Motor Abilities, a test that assesses visual motor, fine motor, and visual-spatial performance in children, indicating an association between GDM and diminished performance in these areas during early childhood (154).

A Swedish cohort study conducted in 2007 evaluated the academic performance of 16-year-old adolescents and reported that children exposed to diabetes during gestation were more likely to experience difficulties in completing compulsory school and achieving lower school marks (28). Similarly, a recent national registry-based cohort study in Denmark, spanning from 1994 to 2001, assessed the academic performance of children born to diabetic mothers. The results indicated that adolescents born to diabetic mothers tended to achieve lower grades than other children, and a history of low birth weight appeared to exacerbate the negative impact of maternal diabetes on the offspring’s school performance (137). Finally, a retrospective cohort study from Denmark assessed the school performance of 3,474 children with intrauterine exposure to T1DM, revealing diminished school test marks in IDMs compared to the general population (155).

### 6.7 Behavioral and psychiatric disorders

Although several studies have linked maternal diabetes to neurodevelopmental disorders such as ADHD and autism, less attention has been given to the association between exposure to high glucose levels and psychiatric disorders, which requires further investigation. Animal models have demonstrated that maternal diabetes, producing excessive pro-inflammatory cytokines that directly affect the CNS, may contribute to developing psychiatric disorders (49).

A recent population-based cohort study conducted in Finland investigated the relationship between various types of diabetes during gestation and future psychiatric disorders in offspring. The study’s results indicated that pre-pregnancy diabetes requiring insulin treatment, followed by T2DM and, to a lesser extent, GDM when combined with an elevated BMI, were associated with an increased risk of mood abnormalities and intellectual impairment in the offspring (43).Furthermore, it has been suggested that elevated peripheral insulin levels can worsen insulin resistance, which has also been linked to depressive phenotypes (156).

Schizophrenia, one of the most severe neuropsychiatric disorders, is believed to result from a complex interplay between genetic and environmental factors. Epidemiological evidence suggests that maternal diabetes may predispose individuals to the future development of schizophrenia (157). Multiple potential pathophysiological pathways have been proposed to explain the increased risk of schizophrenia in the ODMs. These include increased oxidative stress, mitochondrial modifications, altered lipid metabolism, elevated levels of inflammatory cytokines, structural brain abnormalities, and disrupted neurotransmitter metabolism, which has been observed in both the brains of the ODMs and schizophrenia patients (158).

In a recent population cohort study, researchers collected and analyzed data from the ODMs over 39 years, considering the possibility that mental health disorders may manifest later in life. The results revealed a higher risk of developing schizophrenia, behavioral disorders, anxiety, and decreased intellectual ability in this population (139). Another study indicated that 10-year-old children with a history of maternal diabetes during the early gestational period had a higher incidence of hallucinatory experiences (42). Previous research has also demonstrated that gestational diabetes during the first 11 weeks of gestation is associated with an increased risk of developing psychotic experiences during adolescence (159).

Another population-based cohort study conducted in Israel found that maternal diabetes poses a significant risk for the future development of ASD, eating disorders, and obstructive sleep apnea in offspring. Additionally, the study observed that children exposed to maternal hyperglycemia were more prone to developing mental health disorders at a younger age than unexposed children (15).

In a prospective study conducted in 2018, the impact of maternal diabetes and other metabolic complications during pregnancy on communication skills was examined. The study revealed an association between maternal diabetes and suboptimal communication, problem-solving, and socializing skills in the offspring (160).

A longitudinal birth cohort study by Krzeczkowski et al. demonstrated a strong link between maternal diabetes and behavioral and emotional problems in the offspring at 2 years old. Both internalizing and externalizing problems were observed, although these findings appeared to be influenced by confounding factors that could have been prevented, such as maternal diet, depressive symptoms, and socioeconomic status (138).

Furthermore, Kong et al., in their study, proposed that the combination of pre-gestational maternal diabetes and obesity during pregnancy increases the predisposition of children up to 11 years of age to a wide range of psychiatric disorders, including conduct disorders, emotional problems, and social function disorders. The study also reported an elevated prescription rate of psychotropic medication among this group of children (147).

The existing literature does not provide sufficient data to definitively determine whether intrauterine exposure to hyperglycemia predisposes individuals to future psychiatric disorders. Organizing large-scale, well-designed studies to obtain more robust evidence and draw clear conclusions on this matter is essential.

## 7 Conclusion

There is no doubt that intrauterine exposure to hyperglycemic insults can predispose individuals to the development of neurocognitive and behavioral problems in the next generation. This occurs through the induction of an exaggerated peripheral inflammatory response and neuroinflammation in the offspring’s brain. With the global burden of gestational diabetes continuing to rise, it is crucial to identify the potential deleterious effects that this metabolic disturbance has on a child’s health and well-being from early in life.

Numerous studies have attempted to investigate the impact of diabetes on normal brain physiology and its consequences for neurodevelopment in all aspects. However, there is a limited amount of research examining this association, and the existing studies exhibit significant diversity in terms of participant numbers and demographic characteristics, including maternal socioeconomic factors, age, severity, and duration of hyperglycemia. These variations make it challenging to draw clear conclusions.

Despite conflicting findings, emerging evidence suggests that intrauterine hyperglycemia disrupts the neurocircuitry of the offspring, even though the exact underlying mechanisms have not been completely elucidated. To address this knowledge gap and provide accurate results, further research is warranted at both experimental and human levels, involving larger participant cohorts and eliminating confounding factors. The identification of candidate pathophysiological mechanisms by which maternal diabetes alters normal neurodevelopment would be of vital importance, as it could help identify and implement early prevention strategies and targeted therapeutic approaches.

## Author contributions

Conceptualization, VP, CK-G, NI, TB, ZI, and KR; Methodology, VP, CK-G, NI, and KR; Investigation, KR; Writing—original draft preparation, KR; Supervision, CK-G and VP. Review and editing, VP, NI, CK-G, ZI, and TB. All authors contributed to the article and approved the submitted version.
